# Glutathione reductase plays a role in the metabolism of methylmercury degradation in *Rhodotorula mucilaginosa*

**DOI:** 10.1128/spectrum.02395-24

**Published:** 2025-01-16

**Authors:** Yi Guo, Wenlong Deng, Qigui Mo, You Yu, Zhenwang Zhang, Mingjie Wei, Ruiling Tang, Surui Lu, Yanting Su

**Affiliations:** 1 School of Basic Medical Sciences, Xianning Medical College, Hubei University of Science and Technology, Xianning, China; 2 School of Pharmacy, Hubei University of Science and Technology, Xianning, China; 3 Medicine Research Institute and Hubei Key Laboratory of Diabetes and Angiopathy, Hubei University of Science and Technology 271677 , Xianning, China; University of Minnesota Twin Cities, St. Paul, Minnesota, USA

**Keywords:** methylmercury, *Rhodotorula mucilaginosa*, heavy metal, bioremediation, glutathione reductase

## Abstract

Mercury pollution is a kind of heavy metal pollution with great harm and strong toxicity which exists worldwide. Some microorganisms can convert highly toxic methylmercury into inorganic mercury compounds with significantly reduced toxicity. This is an effective means of methylmercury pollution remediation. As a safe microorganism with great potential in the remediation of heavy metal pollution, *Rhodotorula mucilaginosa* has not been studied in the remediation of methylmercury. Here, a *R. mucilaginosa* strain Rm4 with high methylmercury resistance was obtained by wild-strain screening. Its minimal inhibitory concentration and minimum lethal concentration reached 3 and 6 mg/L, respectively. At the same time, Rm4 can also degrade methylmercury. Unlike the traditional microbial methylmercury degradation pathways, *R. mucilaginosa*’s genome does not encode the organomercury lyase gene MerB. However, transcriptomic analysis revealed that the glutathione reductase of *R. mucilaginosa* responds to the methylmercury degradation process. Structural domain analysis and molecular docking experiments suggest that the glutathione reductase of *R. mucilaginosa* has the potential to directly or indirectly participate in methylmercury degradation metabolism. Metabolic indicator tests of engineered strains overexpressing the glutathione reductase encoding gene also support this notion.

**IMPORTANCE:**

The remediation of methylmercury pollution is crucial for environmental health. The ability of *Rhodotorula mucilaginosa* to resist and degrade methylmercury offers a new avenue for bioremediation efforts. Understanding the metabolic pathways involved, particularly the role of glutathione reductase, enhances our knowledge of how *Rhodotorula mucilaginosa* responds to methylmercury and opens up new possibilities for future research in bioremediation strategies.

## INTRODUCTION

Mercury (Hg), located in group IIB of the sixth period of the periodic table, has a remarkably low melting point and is an inert metal with stable chemical properties. This versatile element, discharged into the environment by both natural and anthropogenic sources, manifests primarily in three forms: elemental Hg, inorganic mercury (I_Hg_), and organic mercury (O_Hg_). These forms permeate soil and water systems via atmospheric circulation and dry and wet sedimentation pathways (1). Subsequently, they undergo transformations, culminating in the production of the highly toxic methylmercury (MeHg) through biological and chemical processes (2). Methylmercury, notorious for its ability to bioaccumulate and biomagnify through various trophic levels, poses a significant threat to human health (3–5).

Mercury bioremediation involves the elimination, degradation, or transformation of inorganic or organic mercury present in contaminated media through the catalytic capacity of organisms, enzymes, their biomass compounds, or other derivatives. This process aims to mitigate or eliminate the environmental impacts caused by mercury pollution (4, 6, 7). Microorganisms possess numerous advantages, including robust reproductive capabilities, rapid growth rates, and a highly specific surface area (8). When contrasted with other organisms, microorganisms exhibit greater potential in controlling environmental mercury pollution (9). Microbial remediation of mercury involves mercury-resistant bacteria that can either adsorb mercury pollutants on their cell surfaces or specifically recognize and transport mercury pollutants into cells using transporters such as MerC, MerE, MerF, MerG, MerH, and MerT (10–12). These transporters possess a high specificity for mercury recognition, determined by the amino acid sequence of their metal-binding domain, which grants them an advantage over other heavy metal transporters (11). Once inside the cell, mercury pollutants become sequestered by binding with metabolites such as polyP, mercaptan, and certain trace elements (13–15), effectively reducing the free mercury content in the environment. Therefore, microorganisms can remove mercury compounds from the environment through two mechanisms: surface adsorption and transport absorption. These two principles of remediation have been widely applied and developed into engineering solutions (16, 17). However, they still fall short in addressing the source of highly toxic mercury compounds. Hence, the principle of microbial transformation repair, which aims to convert highly toxic O_Hg_ into less toxic or non-toxic I_Hg_, has garnered significant attention. A small number of mercury-resistant microorganisms can not only transport O_Hg_ pollutants with highly toxic into cells but also resist mercury stress through *mer* operons (18). This process converts mercury from a highly toxic form to a less toxic or non-toxic form (19–21). The *mer* operon is prevalent in both Gram-negative and Gram-positive bacteria. It comprises a set of genes associated with anti-mercury function, encoding proteins involved in mercury transport, transformation, and regulatory mechanisms. Among these, the mercury reductase encoded by the *merA* gene and the organomercury lyase encoded by the *merB* gene play a crucial role in the degradation of mercury and its compounds (22). Narrow-spectrum mercury-resistant bacteria primarily utilize MerA to reduce Hg^2+^ to Hg^0^, whereas broad-spectrum mercury-resistant bacteria can also convert O_Hg_ to Hg^2+^ through MerB in addition to MerA (23, 24). The MerA proteins encoded by the *mer* operons of certain proteobacteria possess functional domains of MerB, enabling them to degrade O_Hg_ (25). Nevertheless, broad-spectrum mercury resistance is not solely reliant on MerA and MerB. Currently, several MerB-dependent microbes that can transform methylmercury have been identified. Wang et al. (8) reported a multifunctional selenite-reducing bacterium capable of reducing Hg^2+^ to Hg^+^ and Hg^0^ while also reducing selenite to selenide, thus possessing both biotransformation and bioaccumulation abilities. This bacterium was able to remove 93.2% of Hg^2+^ from a solution with 40 µg/L mercury pollutants, with the final products being gaseous Hg^0^ and easily separable mercury-containing particles (such as HgSe). Additionally, Cabral et al. (26) isolated a strain of *Pseudomonas putida* from mercury-contaminated soil and sludge in the southern region of Brazil, which lacked *merB* but demonstrated the ability to degrade methylmercury. In a medium with 12 µmol/L methylmercury, this strain removed 29% of methylmercury despite growth inhibition. Under lower methylmercury exposure conditions, it achieved at least 77% and 80% removal efficiencies over a relatively wide range of pH (4–6) and temperature (21°C–25°C). This indicates that there are other unknown pathways for methylmercury degradation in microorganisms.

*Rhodotorula mucilaginosa* was initially isolated and reported from cheese in the 1930s, boasting a nearly century-long history. This fungal species belongs to the *Dikaryota* subdivision within the *Basidiomycetes* class, further categorized under the *Rhizobacteria*, *Microglobularia*, *Claviculariaceae*, and *Rhodotoraceae* families (27). *R. mucilaginosa* exhibits a versatile metabolism capable of utilizing a wide array of carbon sources, including glucose, sucrose, maltose, xylose, waste molasses, corn stalk hydrolysate, potato extract, and various other carbohydrates (28). For nitrogen sources, *R. mucilaginosa* demonstrates versatility, utilizing both inorganic nitrogens like ammonium chloride, ammonium sulfate, and sodium nitrate, as well as organic nitrogen sources such as yeast powder, peptone, and triethanolamine (28). During its growth process, this organism synthesizes a variety of antioxidants, including beta-carotene, torularhodin, and others. Some studies suggest that carotenoids produced by *R. mucilaginosa* may play a role in protecting cells from the effects of intracellular reactive oxygen species induced by ultraviolet light or other abiotic stresses (27, 29, 30). Consequently, *R. mucilaginosa* exhibits robust growth and resistance capabilities even under conditions of nutrient deficiency or external stress.

*R. mucilaginosa* exhibits strong resistance and removal capabilities against cadmium, zinc, nickel, and copper (31, 32). It can adsorb and transport lead, copper, and zinc. Upon heavy metal compound entry into cells, intracellular vacuoles are formed, wherein the concentration of heavy metals exceeds 16% of the medium. *R. mucilaginosa* can also grow in 80 mg/L HgCl and adsorb 69.9 mg/g of Hg^2+^ (33). Only a small fraction of *R. mucilaginosa* strains are conditionally pathogenic (34), while the majority can serve as safe industrial strains for producing lipids, proteins, and other substances (35). Consequently, compared to existing microorganisms capable of degrading methylmercury, *R. mucilaginosa* boasts higher safety credentials. This suggests that *R. mucilaginosa* holds significant application potential for heavy metal adsorption and conversion processes. Currently, there are no published studies on the absorption and degradation of highly toxic organic mercury by *R. mucilaginosa*. In our study, we discovered that *R. mucilaginosa* cells possess strong methylmercury tolerance and degradation abilities, despite the absence of MerB, which is encoded by a broad-spectrum anti-mercury gene. Instead, we found that its glutathione reductase plays a role in the degradation metabolism of methylmercury. These findings provide a foundation for exploring a new metabolic mechanism of methylmercury degradation in fungi and potentially advance the application of *R. mucilaginosa* in the field of methylmercury pollution remediation.

## MATERIALS AND METHODS

### Strains, plasmids, and chemicals

All of the yeast strains and plasmids used in this study are listed in Tables 1 and 2. The primers used for strain and plasmid construction or qPCR are listed in Table S1. *Escherichia coli* DH5*α* (*supE44* Δ*lacU169* [*φ80lacZ*Δ*M15*] *hsdR17 recA1 endA1 gyrA96 thi-1 relA1*) was used as a general host for plasmid construction and propagation. *R. mucilaginosa* Rm1–Rm4 were used to verify tolerance of methylmercury, and Rm4 and its derivative strain were used to degrade methylmercury and verify the related gene. Plasmid pYE-TNC and its derivative plasmid, which contains the expression cassette of resistance gene of geneticin (G418), were used to develop genetic manipulation methods and overexpress glutathione reductase in *R. mucilaginosa*. A Luria-Bertani medium containing 10 g/L tryptone, 5 g/L yeast extract, and 10 g/L NaCl was used for *E. coli* cell cultivation; when necessary, 100 µg/mL of ampicillin was added to the medium. A YPD medium containing 10 g/L yeast extract, 20 g/L tryptone, and 20 g/L dextrose was used for yeast cell cultivation, and 25 µg/mL G418 was added when required. The chemical methylmercuric chloride was purchased from Dr. Ehrenstorfer (Germany).

**TABLE 1:** Yeast strains used in this study

Strains	Description	Source
YS58	MATa flo1 ura3-52 leu2-3,112 his4-519 trp1-789	Reference (36)
Rm1	*R. mucilaginosa* with methylmercury resistance	This study
Rm2	*R. mucilaginosa* with methylmercury resistance	This study
Rm3	*R. mucilaginosa* with methylmercury resistance	This study
Rm4	*R. mucilaginosa* with high methylmercury resistance	This study
Rm4-pYE-TNC	Rm4 containing plasmid pYE-TNC	This study
Rm4-GLR1	Rm4 containing plasmid pYE-TNC-GLR1	This study

**TABLE 2:** Plasmids used in this study

Plasmid	Description	Source
YEp352	*E. coli*-yeast shuttle vector, Ampr and *URA3*	Reference (37)
pGMZC	YEp352, P*_GAL1_-mazF*-T*_AOX1_* and P*_TEF1_-zeoR*-T*_CYC1_*	Reference (38)
pYE-TNC	YEp352 with P*_TEF1_*-Neo^r^-T*_CYC1_*	This study
pYE-TNC-GLR1	pYE-TNC with P*_TEF1_-GLR1*-T*_SUP4_*	This study

### Isolation and screening of strains

Rm1–Rm4 were obtained from soil and water samples from heavy metal-polluted areas. The YPD medium with 200 µg/mL chloramphenicol and 1 mg/L methylmercuric chloride was added to the centrifuge tube containing the collected sample and incubated at 28°C for 3 days. The appropriate dilution ratio was selected to dilute the culture. The culture was uniformly coated on YPD solid medium containing chloramphenicol and incubated at 28℃ for 3 days. Red single colonies were selected for internal transcribed spacer (ITS) sequence amplification and sequencing.

### Determination of the minimal inhibitory concentration and minimum lethal concentration to methylmercury

The assay was performed in a 96-well microplate (Costar, Kennebunk, USA). Each well was flled with YPD minimal medium, yeast biomass (living or dead), and a heavy metal suspension from stock solutions. Stock solutions were prepared using analytical-grade methylmercuric chloride. The treatments included a positive control (living yeast biomass and YPD medium), a negative control (dead yeast biomass and YPD medium), the reagent control, and the test solutions. Fungal growth was evaluated after a 24 h incubation period at 28°C using a microplate reader (BioTek Synergy HTX) with a 600 nm flter. The minimal inhibitory concentration (MIC) was considered as the lowest concentration of methylmercuric chloride that completely inhibited the detectable growth of the test strain in the wells. The culture solution was coated on YPD solid plate and cultured at 28°C for 48 h. The minimum lethal concentration (MLC) was considered the lowest concentration of methylmercuric chloride that completely kills the test strain in the wells.

### Carotenoid measurement

About 5 mL broth was centrifuged at 12,000 rpm for 1 min and washed with distilled water. Cell pellets were mixed with 1.8 mL hydrochloric acid (3 M), fully shaken and soaked for 30 min, and then incubated in a boiling water bath for 8–9 min. After observing that the cells became flocculent, they were immediately cooled down in an ice bath. The cell pellets after distilled water washing were extracted with acetone, then shaken and extracted for 15 min in the dark until the cells were colorless. They were centrifuged at 8,000 rpm for 5 min, and the supernatant was taken. Carotenoids were measured at a wavelength of 474 nm using a UV spectrophotometer. The calculation of carotenoid content was as described in the literature (39).

### Analysis of MeHg in different matrixes

As for measurement of MeHg in culture medium and eluent, acidified liquid sample samples were distilled according to a standard operating procedure modified after EPA method 1630 (40) and analyzed by a MERX automated alkyl Hg system (Brooks Rand Labs, USA). For the analysis of MeHg in cells, samples were digested by 25% KOH/CH3OH (wt/vol), ethylated, and finally analyzed by automated alkyl Hg system following the method modified after EPA method 1630 (41).

### Transcriptome analysis (RNA-seq)

Rm4 was grown in YPD medium at 28°C and 220 rpm for 48 h. After adding methylmercury to the Rm4-HgCH_3_ group at a final concentration of 3 mg/L, both the experimental and control groups were co-incubated at 28°C with shaking at 220 rpm for 5 h. After centrifugation at 5,000 rpm to collect the cells, the cells were washed with 3 mL of distilled water and centrifuged. This washing process was repeated twice, and the final bacterial cells obtained were used for transcriptome analysis. The subsequent procedures for RNA sequencing were conducted by Anoroad (Beijing, China). Clean data were *de novo* assembled by the Trinity software program, version 2. The genes with fold changes >1.5-fold were functionally classified using the Munich Information Center for Protein Sequences’ FunCat. There were three parallel settings for each sample.

### Construction of plasmids and yeast strains

General DNA manipulations in *E. coli* was performed according to the standard methods. Genetic manipulation in *R. mucilaginosa* was conducted as described in the Results and Discussion section. For construction of plasmid, plasmid pGMZC and the genomes of YS58 and Rm4 were used as templates. The sequences of expression cassette of the resistance gene was obtained by PCR amplification. The sequences of *TEF1* promoter and gene encoding glutathione reductase with leavage sites were also obtained by PCR amplification. Restriction endonuclease and T4 ligase were used to construct plasmid pYE-TNC and pYE-TNC-GLR1. To verify function of glutathione reductase in methylmercury metabolism, pYE-TNC-GLR1 with *GLR1* expression cassette was overexpressed in Rm4 to generate strain Rm4-GLR1.

### Preparation and transformation of *R. mucilaginosa* competent cells

*R. mucilaginosa* cells were cultured in 5 mL YPD medium at 30°C with shaking at 200 rpm overnight. Subsequently, 100 µL of the overnight culture was inoculated into 100 mL YPD medium and incubated at 30°C with shaking at 200 rpm for 8 h. Cells were harvested by centrifugation at 5,000 × *g* for 5 min at 4°C. The cell pellet was washed with 100 mL pre-chilled sterile water, followed by centrifugation at 5,000 × *g* for 5 min. The cells were resuspended in 5 mL pre-chilled 1 mol/L sorbitol solution and centrifuged again at 5,000 × *g* for 5 min. The final pellet was resuspended in 1 mL pre-chilled 1 mol/L sorbitol solution. A 200 µL aliquot of the resuspended cells was mixed with 10 µg of plasmid DNA (in a volume not exceeding 10 µL) and transferred into a pre-chilled 0.2 cm electroporation cuvette. Electroporation was performed using a Bio-Rad electroporator with the following parameters: 50 µF, 100–129 Ω, and 1.5 kV, with a pulse duration of 4–5 ms. Immediately after electroporation, 1 mL of pre-chilled sorbitol solution was added to the cuvette, and the mixture was transferred to a 1.5 mL microcentrifuge tube. Cells were recovered at 30°C for 3 h and then centrifuged at 5,000 × *g* for 5 min. The pellet was resuspended in 50 µL of sterile water and spread on YPD agar plates containing the appropriate antibiotic. Plates were incubated at 30°C for 48 h.

### Quantification of transcriptional levels of the target genes by qRT-PCR

Yeast cells were cultured in YPD at 30°C and 200 rpm for the required time. The RNAprep Pure Cell/Bacteria Kit (Tiangen, Beijing, China) was used to extract the total RNA according to the manufacturer’s instructions. The transcription levels of target genes were analyzed by quantitative real-time PCR (qRT-PCR) using the Quant OneStep qRT-PCR Kit (SYBR Green) (Tiangen) and LightCycler 96 System (Roche, Switzerland) with primers shown in Table S1. Data were collected and processed by the second-derivative maximum method of the LightCycler 96 software, version SW1.1. Transcription levels of target gene were determined with housekeeping gene *ACT1* as the control to normalize different samples. All relative transcription levels of the target gene in the experimental group were normalized to the target gene levels in the control group to account for differences between samples.

## RESULTS AND DISCUSSION

### Screening wild strains with high methylmercury resistance

**Fig 1:**
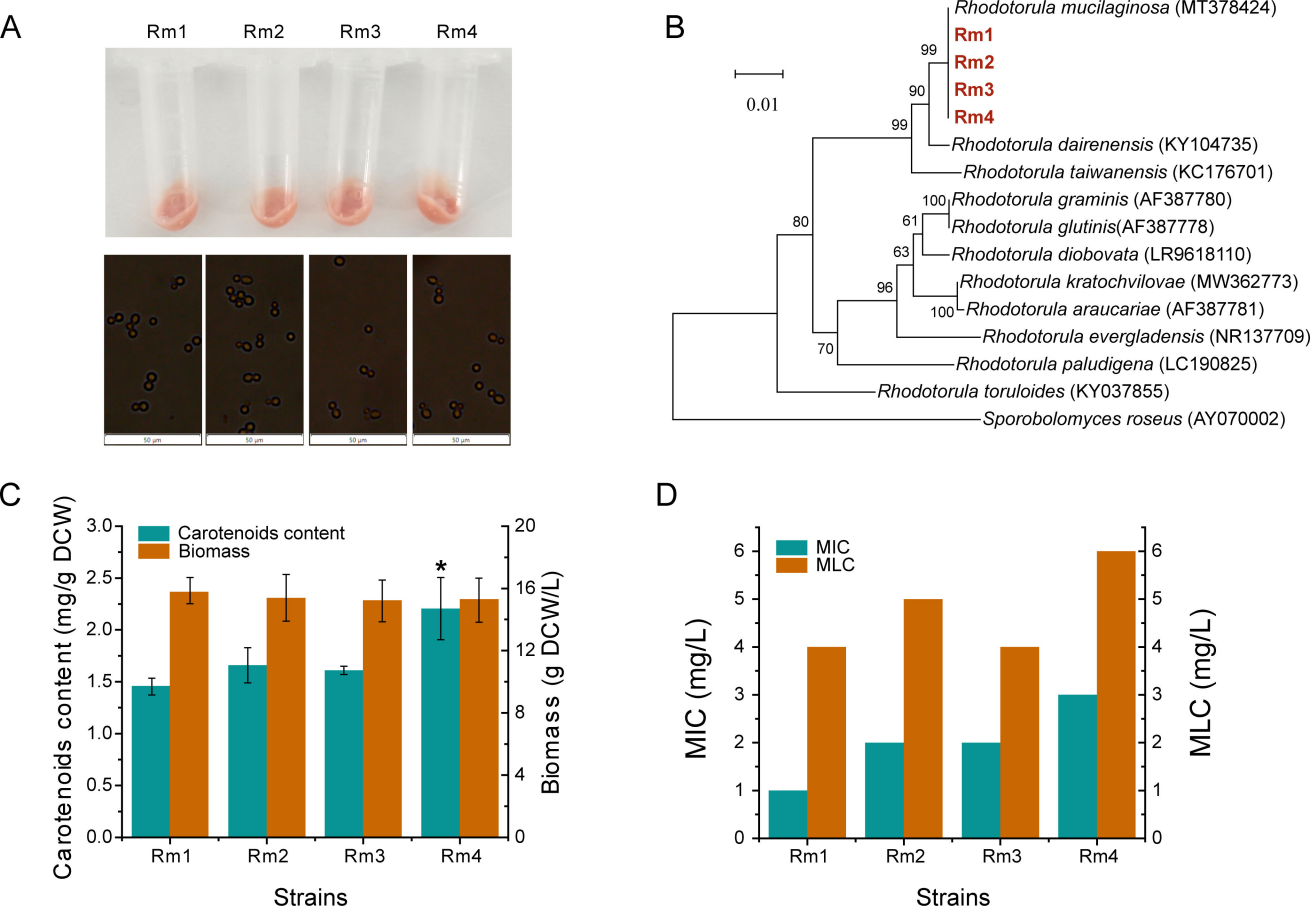
Screening of wild *R. mucilaginosa* strains and resistance to methylmercury. (**A**) Micrograph of the wild strains. (**B**) Evolutionary tree. (**C**) Carotenoid content and biomass of wild *R. mucilaginosa* strains were cultivated in 3 mL of YPD at 28°C and 220 rpm for 24 h in a test tube. (**D**) MIC and MLC of methylmercury on *R. mucilaginosa*. Yeast cells were cultivated in 3 mL of YPD and different concentrations of methylmercury at 28°C and 220 rpm for 24 h in a test tube. Data are presented as the means of the results from three replicate experiments. Error bars represent standard deviations. Student’s *t*-test was used for statistical analysis. *P < 0.05.

In the initial phase, four red strains with resistance to methylmercury were obtained through field sampling and screening (Fig. 1A). A molecular phylogenetic analysis based on the ITS gene nucleotide sequence revealed that these four strains belonged to the genus *Rhodotorula* and exhibit high sequence similarity with *Rhodotorula mucilaginosa* (*MT378424*). Combined with several of these traits, all of them were identified as *R. mucilaginosa* strains and named as Rm1–Rm4 (Fig. 1B). There was no significant difference in biomass between these four wild strains. Carotenoid contents in Rm1–Rm4 were 1.46, 1.66, 1.61, and 2.21 mg/g, respectively. Among these strains, Rm4 had the highest carotenoid synthesis (Fig. 1C). It implied that Rm4 might have stronger methylmercury resistance than the other three strains. Because carotenoids synthesis in *R. mucilaginosa* was positively correlated with its own robustness, which was also demonstrated by the MIC and MLC of methylmercury for these four wild strains. For Rm4, MIC was 3 mg/L and MLC is 6 mg/L, both higher than Rm1–Rm3 (Fig. 1D). Compared to other species, the methylmercury tolerance of *R. mucilaginosa* has significant advantages. For some other microorganisms, even microgram-level concentrations of methylmercury can significantly affect their growth (8, 26, 42).

### Removal and degradation of methylmercury by Rm4

**Fig 2:**
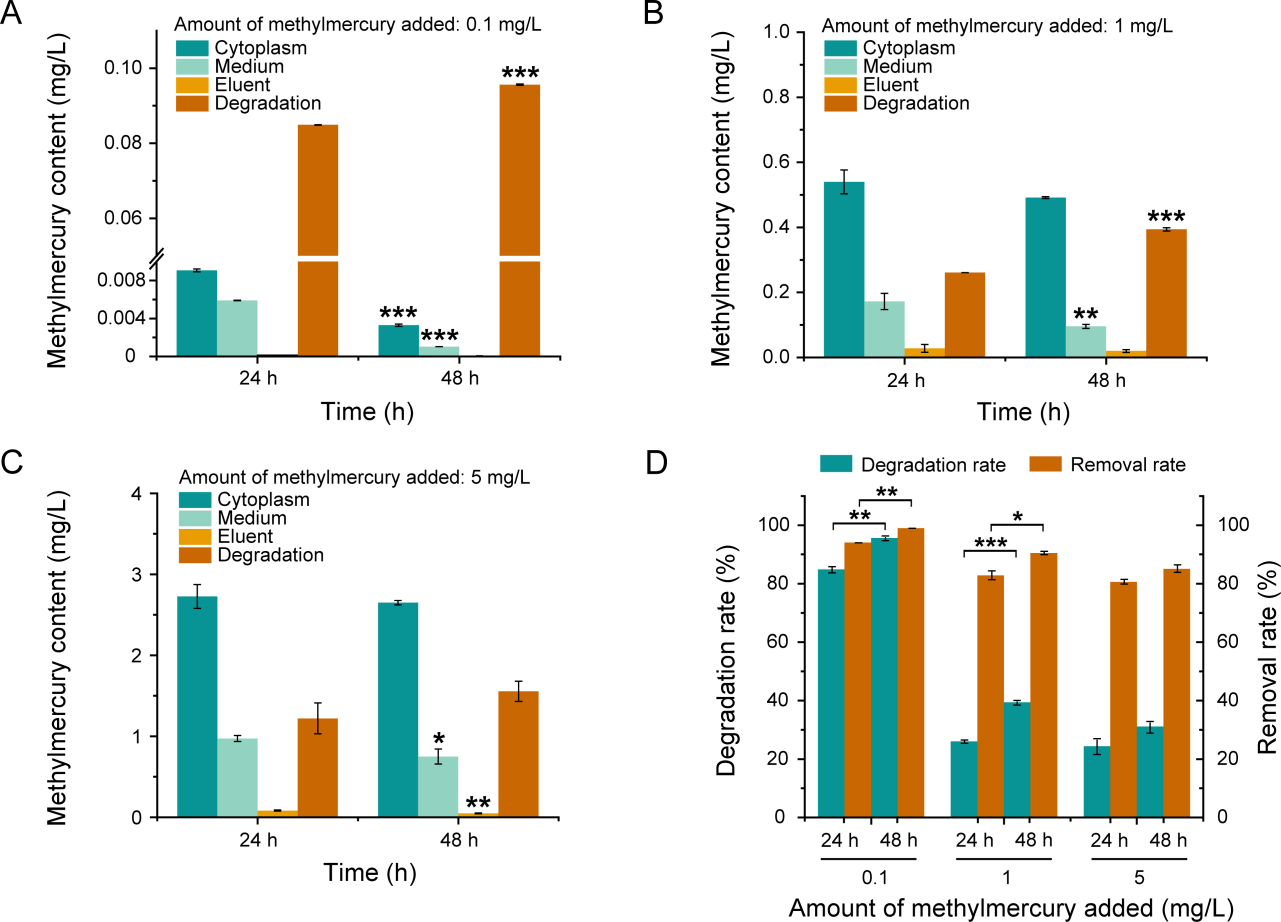
Degradation capacity of *R. mucilaginosa* under different concentrations of methylmercury. (**A**) Methylmercury degradation capacity at 0.1 mg/L methylmercury. (**B**) Methylmercury degradation capacity at 1 mg/L methylmercury. (**C**) Methylmercury degradation capacity at 5 mg/L methylmercury. (**D**) Methylmercury degradation rate and removal rate of *R. mucilaginosa* under different conditions. Yeast cells were cultivated in 5 mL of YPD and different concentrations of methylmercury at 28°C and 220 rpm for 24 h in a test tube. Then different concentrations of methylmercury were added and incubated for 24 or 48 h. Data are presented as the means of the results from three replicate experiments. Error bars represent standard deviations. Student’s *t*-test was used for statistical analysis. *P < 0.05, **P < 0.01, ***P < 0.001.

When incubated with various concentrations of methylmercury for 24 or 48 h, Rm4 demonstrated strong capabilities in the removal and degradation of methylmercury. For example, when incubated with 0.1 mg/L methylmercury, significant degradation was observed after 24 h, with minimal methylmercury remaining in the cells and the medium (Fig. 2A). After 48 h, the degradation rate of methylmercury reached 98.97% (Fig. 2D). In higher concentrations (1 and 5 mg/L), while the removal efficiency exceeded 80%. The degradation amounts after 24 h reached 0.26 and 1.22 mg/L and increased to 0.39 and 1.55 mg/L after 48 h (Fig. 2B and C). The degradation rates varied among the different concentrations, with the 0.1 mg/L group showing a rate of 0.0035 mg/L/h, while the rates for 1 and 5 mg/L were 0.011 and 0.051 mg/L/h, respectively. The above experimental results indicate that Rm4 exhibits excellent removal and degradation capabilities under different concentrations of methylmercury, particularly at low concentrations (0.1 mg/L). In higher concentrations of methylmercury (1 and 5 mg/L), the degradation rate significantly decreases. This may be related to the increased substrate load. At the same time, the higher concentration of methylmercury can also impose a greater metabolic burden on the cells (43). Specifically, elevated concentrations of methylmercury may inhibit the physiological activities of the cells, thereby affecting the degradation rate.

### Effects of methylmercury exposure on the transcriptome of Rm4

**Fig 3:**
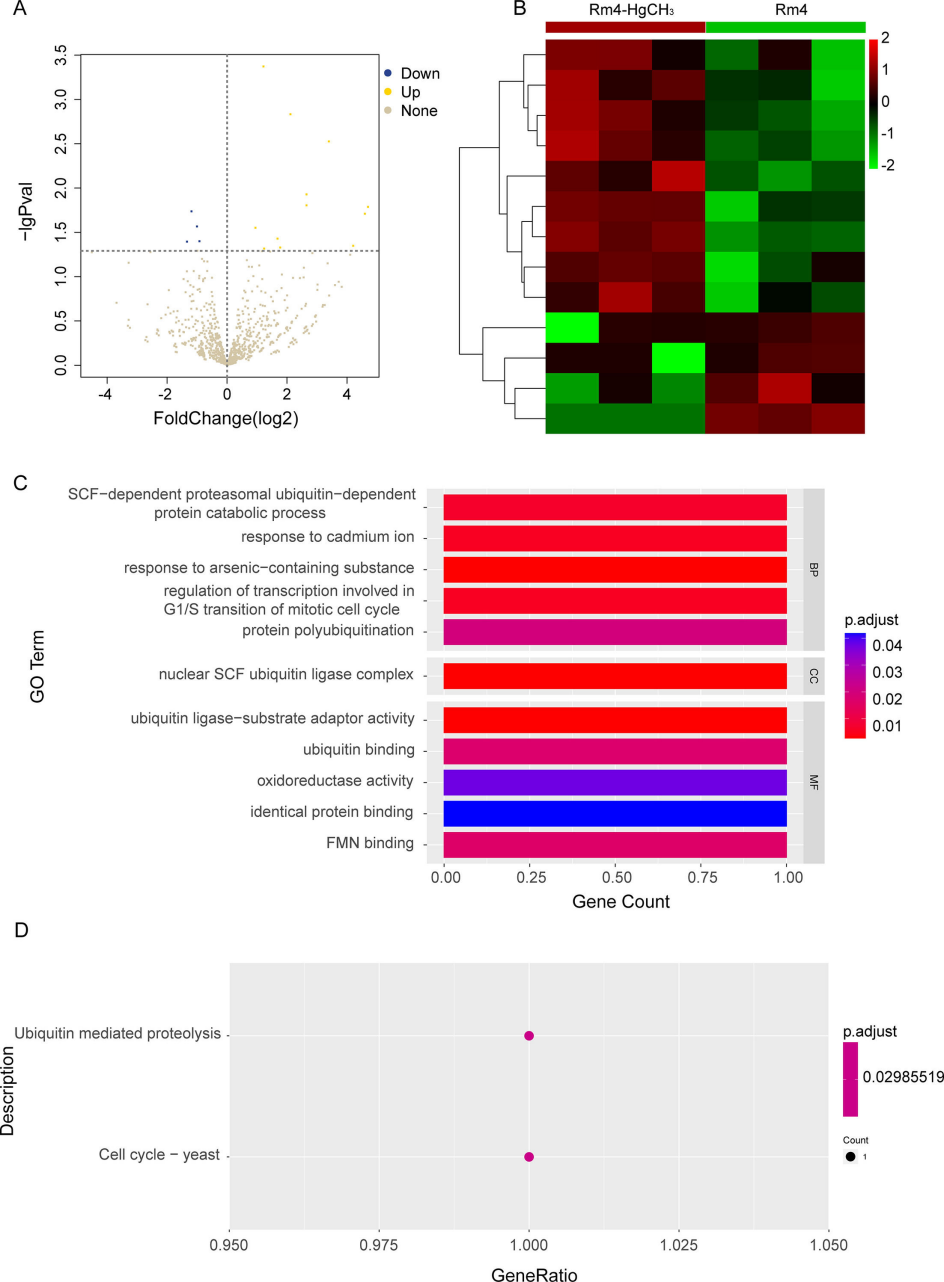
Characterization of the transcriptome data set in the control and methylmercury-treated cultures. (**A**) Volcano plot of differentially expressed genes at a *q* value of <0.05 level with |log2 fold change| of ≥0.5. (**B**) Differential gene cluster map. The heatmap of the enrichment of the differentially expressed genes of Rm4 with Rm4 exposed to methylmercury. The legend of the color bar displays the log2 values of the fold change of each replicate of each gene, from −2 to 2. (**C**) GO enrichment analysis. (**D**) Kyoto Encyclopedia of Genes and Genomes enrichment analysis. The enrichment was demonstrated in three successful repetitions.

The transcription levels of 13 genes in the Rm4 which was exposed to methylmercury were changed (Fig. 3A), of which 9 genes were upregulated and 4 genes were downregulated (Fig. 3B; Table S2). According to the Gene Ontology (GO) enrichment analysis of differential genes, the number of differential genes in each item was analyzed by a hypergeometric test, and the significantly enriched GO items were found (with *Q* < 0.05 as the threshold, and the GO term satisfying this condition as the significantly enriched GO entries) (Fig. 3C). In the biological process category, the process of SCF-dependent proteasomal ubiquitin-dependent protein catabolic, response to cadmium ion, response to arsenic-containing substance, and regulation of transcription involved in G1/S transition of mitotic cell cycle were enriched. In the molecular functional categories, five entries were shown, including ubiquitin ligase-substrate adaptor activity, ubiquitin binding, oxidoreductase activity, identical protein binding, and FMN binding. Gene encoding glutathione reductase was upregulated, which was closely related to oxidative stress. The nuclear SCF ubiquitin ligase complex was enriched in the cellular component. Taking *Q* < 0.05 as the standard, the enrichment analysis of each pathway in Kyoto Encyclopedia of Genes and Genomes (KEGG) was carried out by a hypergeometric test (44), and the significant enrichment of the pathway from differentially expressed genes was found. In the Rm4 which was exposed to methylmercury, significant changes have taken place in two aspects (Fig. 3D): ubiquitin-mediated proteclysis and cell cycle in yeast (45). In the cell cycle pathway, the gene MET30, which co-codes for the SCF-MET30 E3 ubiquitin ligase complex, is significantly upregulated. (46). Met30p controls cell cycle function, sulfur metabolism, and methionine biosynthesis as part of the ubiquitin ligase complex (47). It also interacts with and regulates Met4p, which localizes within the nucleus. Dissociation of Met30p from the SCF complex, in response to cadmium stress, is regulated by Cdc48p (48). The results of GO enrichment and KEGG enrichment indicated that the transcription levels of the Rm4, which was exposed to methylmercury, were changed in multidimension not only in molecular function and metabolic processes but also in the cell cycle.

### Prediction of closely related pathways of methylmercury degradation metabolism in *R. mucilaginosa*

**Fig 4:**
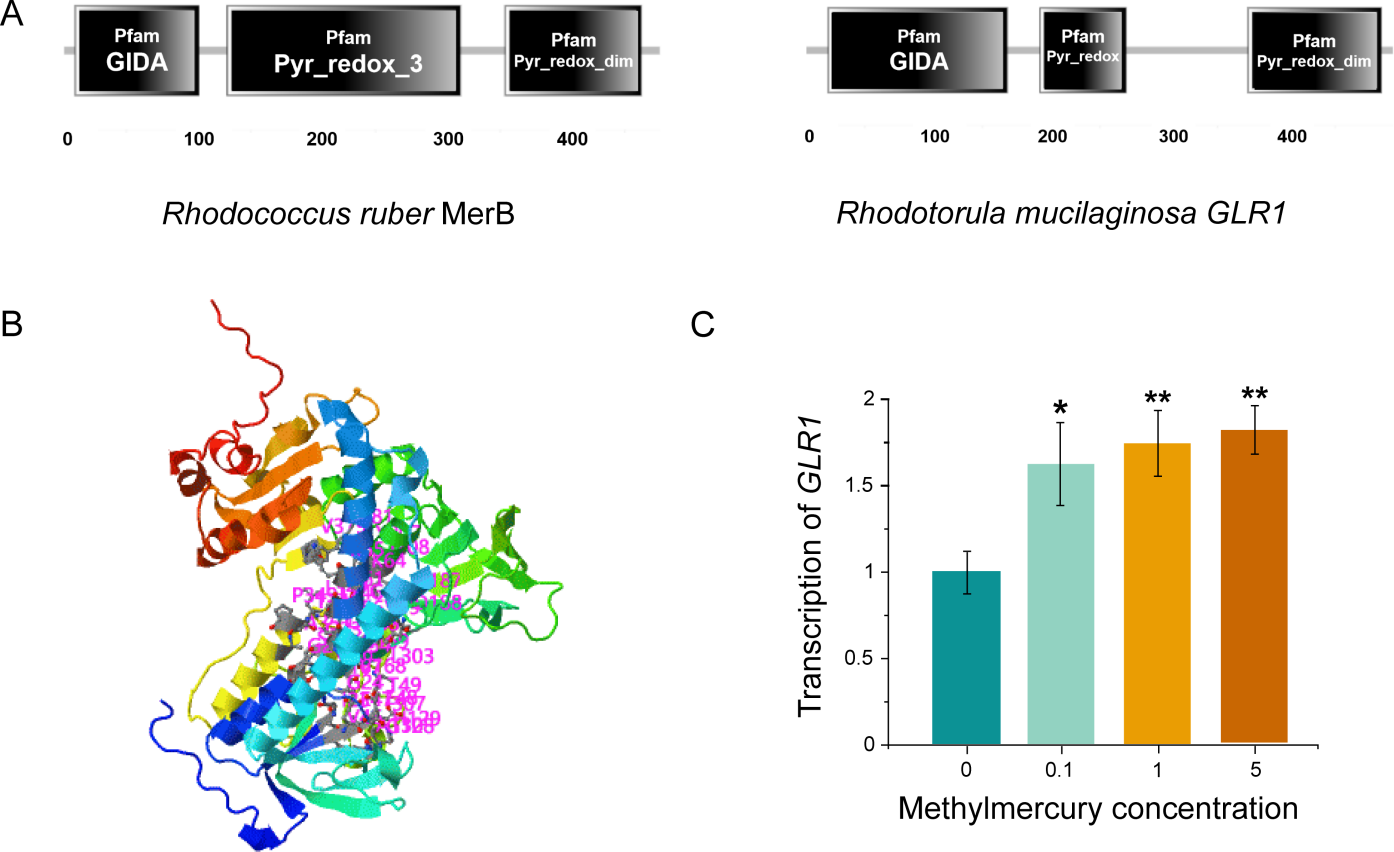
Predicted structure of GLR1 and its response to methylmercury stress. (A) Functional domains of MerB in *Rhodococcus ruber* and glutathione reductase in *R. mucilaginosa*. (B) Possible binding site of methylmercury to glutathione reductase. (C) Effect of methylmercury on GLR1 transcription. Yeast cells were cultivated in 5 mL of YPD at 28 °C and 220 rpm for 24 h in a test tube. Then 1 mg/L methylmercury were added and incubated for 24h. Data were presented as the means of the results from three replicate experiments. Error bars represented standard deviations. The Student t test was used for statistical analysis (*P < 0.05, **P < 0.01).

For the first time, previous experiments demonstrated that *R. mucilaginosa* could degradate methylmercury. However, this metabolic pathway of degradation is still unclear. Transcriptomic GO analysis brings glutathione reductase into focus. Sequence homology comparison indicates that the glutathione reductase in *R. mucilaginosa* has about 70% similarity to the mercury reductase MerA (Fig. S1), which includes critical active regions for mercury metabolism. This aligns with previous reports concluding the homology between glutathione reductase and MerA (25). However, no enzyme protein sequence similar to the broad-spectrum mercury metabolic enzyme MerB was found in *R. mucilaginosa*, which contradicts our earlier conclusion that this yeast possesses the capability to convert methylmercury. This suggests the existence of a methylmercury transformation pathway in *R. mucilaginosa* that does not rely on MerB. Additionally, a very interesting finding was that functional domain structure analysis revealed a similar distribution of functional domains between the glutathione reductase of *R. mucilaginosa* and MerB (Fig. 4A). The protein structure model of glutathione reductase was constructed by AlphaFold, and 38 possible amino acid sites were obtained by docking with small methylmercury molecules (Fig. 4B; Table S3). In addition, the relative expression of glutathione reductase in cells incubated with methylmercury also increased significantly, reaching more than 1.6 times that of the control group. (Fig. 4C). These results indicated that glutathione synthase was closely related to the pathway of methylmercury degradation in *R. mucilaginosa*. However, the function of glutathione reductase in *R. mucilaginosa* has been confirmed under Cd and As stress (48). It significantly enhances glutathione reductase activity in response to both metals. Additionally, it increases catalase activity. This suggests that the primary role of glutathione reductase here is to mitigate oxidative stress induced by abiotic stressors. Zheng et al. (49) also revealed from omics perspective the role of glutathione reductase in *R. mucilaginosa* in alleviating oxidative stress responses. However, to date, there have been no studies regarding the function of glutathione reductase in *R. mucilaginosa* in methylmercury metabolism.

### Genetic manipulation methods and molecular tools of *R. mucilaginosa*

**Fig 5:**
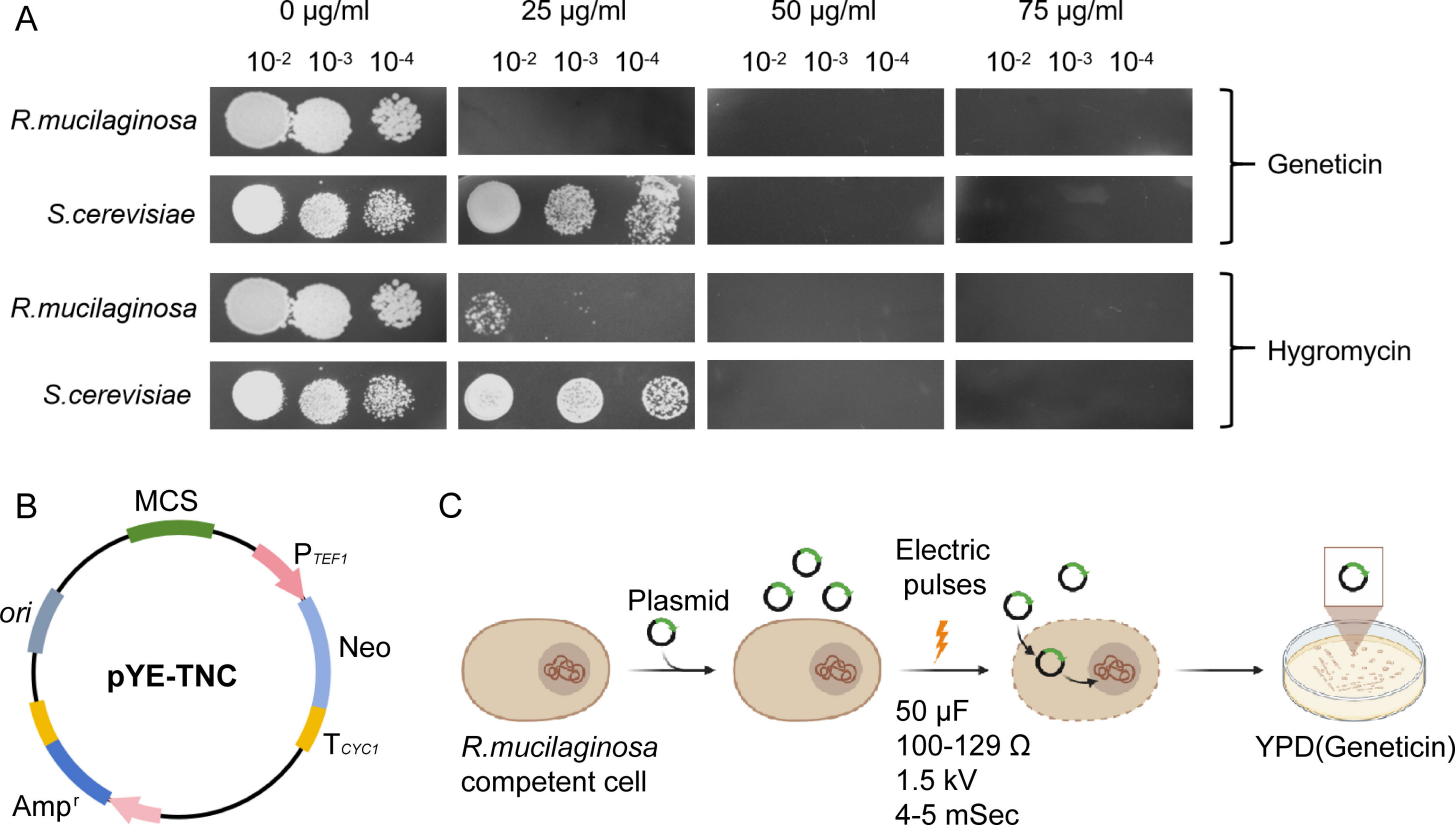
Genetic manipulation methods and molecular tools of *R. mucilaginosa*. (**A**) Screening of antibiotics: for environmental considerations, the resistance testing of two different strains against the same antibiotic at the same concentration was conducted on the same plate, which might result in a slight overlap in the images. Additionally, since plates with 0 µg/mL geneticin or cycloheximide are equivalent to YPD plates without antibiotics, the growth of the two different strains on the YPD plates with 0 µg/mL geneticin and 0 µg/mL cycloheximide was captured using the same image. (**B**) Map of the overexpressed plasmid. (**C**) Parameters of electric shock transformation method of *R. mucilaginosa*.

The genetic manipulation method of *R. mucilaginosa* has not been reported at present. To verify the function of glutathione reductase in the cell, it is necessary to develop molecular tools and transformation methods that can be used for enzyme protein expression. The first step was to screen for antibiotics which could be used as screening markers. Geneticin (G418) prevents protein synthesis by interfering with ribosomal function, leading to toxicity in eukaryotic cells (50). Bleomycin acts by binding to DNA and inducing nucleic acid strand breaks, also causing widespread toxicity in eukaryotic cells (51). Therefore, these two antibiotics were chosen to test on *R. mucilaginosa*. G418 of 25 µg/L and hygromycin of 50 µg/L could effectively inhibit the growth of *R. mucilaginosa* (Fig. 5A). Both antibiotics can be used as resistance markers. The plasmid pYE-TNC with G418 resistance gene expression box was constructed using the *E.coli*-yeast shuttle plasmid pYE as the plasmid skeleton (Fig. 5B). According to the preparation method of the sensor cells of *Saccharomyces cerevisiae*, the sensor cells of *R. mucilaginosa* were prepared. After mixing the sensor cells with pYE-TNC plasmid, the electroporation transformation was carried out by using a Bio-Rad electrotrometer with a shock pulse setting of 50 µF, 100–129 Ω, and 1.5 kV for 4–5 ms (Fig. 5C). *R. mucilaginosa* cells transformed with the pYE-TNC plasmid could grow on YPD solid medium supplemented with G418. This means that all functional elements on the plasmid, as well as the method of electroshock transformation, are suitable for *R. mucilaginosa*. The pYE-TNC can be used for the expression of the target enzyme protein.

### Effects of glutathione reductase overexpression on methylmercury tolerance and degradation in *R. mucilaginosa*

**Fig 6:**
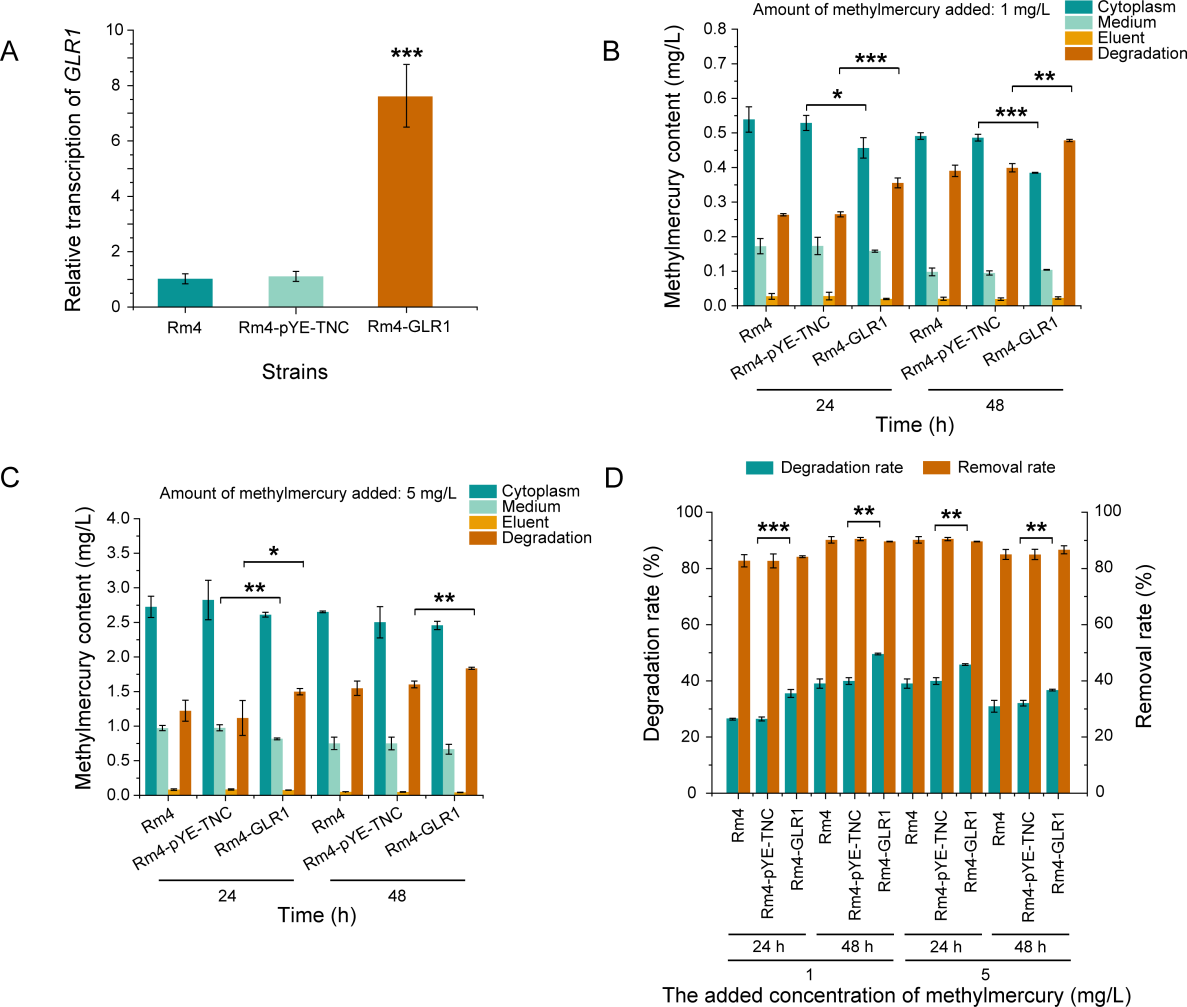
Effect of overexpression of *GLR1* on methylmercury degradation. (**A**) Relative transcription levels of *GLR1*. (**B**) Methylmercury degradation capacity at 1 mg/L methylmercury. (**C**) Methylmercury degradation capacity at 5 mg/L methylmercury. (**D**) Methylmercury degradation rate and removal rate of *R. mucilaginosa* under different conditions. Yeast cells were cultivated in 5 mL of YPD and different concentrations of methylmercury at 28°C and 220 rpm for 24 h in a test tube. Then different concentrations of methylmercury were added and incubated for 24 or 48 h. Data are presented as the means of the results from three replicate experiments. Error bars represent standard deviations. Student’s *t*-test was used for statistical analysis. *P < 0.05, **P < 0.01, ***P < 0.001.

To investigate the function of glutathione reductase in methylmercury metabolism, GLR1, which codes for glutathione reductase, was overexpressed using the plasmid pYE-TNC-GLR1 in Rm4-GLR1 (Fig. 6A). Control strain Rm4-TGC, which was transferred with the pYE-TNC, was also constructed. From all aspects related to methylmercury, Rm4 and Rm4-pYE-TNC showed no difference (Fig. 6B and C), which proved that plasmid pYE-TNC had no effect on methylmercury-related functions. Compared to Rm4-pYE-TNC, Rm4-GLR1 exhibited significantly increased tolerance to methylmercury, with the MIC and MLC of methylmercury for Rm4-GLR1 increased to 5 and 7 mg/L, respectively (Table S4). After incubation with 1 mg/L methylmercury for 24 h and 48 h, the degradation of methylmercury in Rm4-GLR1 reached 0.36 mg/L and 0.50 mg/L, respectively, which was 38% and 24% higher than that in the control strain Rm4-pYE-TNC. For Rm4-GLR1, the degradation rates reached 0.015 mg/L/h in the previous 24 h and 0.0059 mg/L/h within 24–48 h. These were higher than the rates of Rm4-pYE-TNC (0.011 and 0.0056 mg/L/h). After 5 mg/L methylmercury incubation for 24 and 48 h, the degradation amount of methylmercury in Rm4-GLR1 reached 1.50 and 1.84 mg/L, which were 34% and 14% higher than those in Rm4-pYE-TNC. For Rm4-GLR1, the degradation rates reached 0.015 mg/L/h in the previous 24 h and 0.0059 mg/L/h within 24–48 h. Although the rate of degradation increased, the rate of removal had no change (Fig. 6D). This indicates that glutathione reductase may enhance the ability of cells to resist oxidative stress, allowing *R. mucilaginosa* cells to maintain vitality for an extended period under high concentrations of methylmercury, thereby sustaining higher metabolic activity, which significantly increases the degradation of methylmercury. Furthermore, considering that glutathione reductase has numerous potential binding sites and functional domains related to mercury metabolism, it may also be directly involved in the catabolism of methylmercury. However, direct evidence supporting this hypothesis is still lacking. Nevertheless, the experimental results of this study can still indicate that glutathione reductase may play a role in the tolerance and degradation of methylmercury in *R. mucilaginosa*.

### Conclusion

In summary, this experiment first obtained a strain of *R. mucilaginosa* Rm4 with strong methylmercury tolerance through the screening of wild yeast. Through methylmercury incubation experiments, it was found that Rm4 has strong methylmercury removal and transformation capabilities. Transcriptome sequencing analysis of Rm4 and Rm4 exposed to methylmercury revealed a new important target gene *GLR1*. By overexpressing *GLR1* in Rm4, its potential role in the methylmercury tolerance and degradation process of *R. mucilaginosa* was verified. However, the specific mechanism of action and the degradation products of methylmercury still require further research. Based on the analysis of differentially expressed genes, it was discovered that the metabolic pathways in ubiquitin mediated proteolysis and cell cycle. The discovery of the above crucial target gene and pathways is helpful to understand the *R. mucilaginosa* response to methylmercury stress and degradation process of methylmercury. It suggests that further mining the key information of the transcriptome is of great significance for an in-depth elaboration of the new methylmercury degradation metabolism pathway in *R. mucilaginosa* and molecular regulation mechanism, which can hopefully guide the efficient degradation of methylmercury in *R. mucilaginosa*.

## SUPPLEMENTAL MATERIAL

Supplemental material spectrum.02395-24-s0001.docx Tables S1 to S4; Fig. S1.

ASM does not own the copyrights to Supplemental Material that may be linked to, or accessed through, an article. The authors have granted ASM a non-exclusive, world-wide license to publish the Supplemental Material files. Please contact the corresponding author directly for reuse.
