# Supplementary material for: Glutathione reductase plays a role in the metabolism of methylmercury degradation in Rhodotorula mucilaginosa
Source: Microbiol Spectr. 2025 Jan 16;13(2):e02395-24. doi: 10.1128/spectrum.02395-24 (PMC11792481; doi:10.1128/spectrum.02395-24)
Supplement: Supplemental material — Tables S1 to S4; Fig. S1. [file spectrum.02395-24-s0001.docx]

Supplementary information for:

**Glutathione reductase plays a role in the metabolism of methylmercury degradation in *Rhodotorula mucilaginosa***

Yi Guo ^1#^, Wenlong Deng^3#^, Qigui Mo^2^, You Yu^1^, Zhenwang Zhang^2^, Mingjie Wei^3^, Ruiling Tang^1^, Surui Lu^1^[[1]](#footnote-2)^*^, Yanting Su^1^[[2]](#footnote-3)^*^

1. School of Basic Medical Sciences, Xianning Medical College, Hubei University of Science and Technology, Xianning 437100, China.
2. Medicine Research Institute & Hubei Key Laboratory of Diabetes and Angiopathy, Hubei University of Science and Technology, Xianning 437100, China.
3. School of Pharmacy, Hubei University of Science and Technology, Xianning 437100, China.

**Running title:**

Methylmercury degradation in yeast

* Corresponding author:

Surui Lu, Hubei University of Science and Technology, No. 88 Xianning avenue, Xianning 437100, China. Tel: +86-18810916270; Email: 942658872@qq.com; 20231017@hbust.edu.cn.

Yanting Su, Hubei University of Science and Technology, No. 88 Xianning avenue, Xianning 437100, China. Tel: +86-13554376312; Email: 2014202040134@whu.edu.cn.

Table S1. Primers used in this study

| primers | sequences (5′ - 3′) | target |
| --- | --- | --- |
| R01 | gagctcggtaccGAATCCTTACATCACAC | P*_TEF1_* |
| R02 | gagtcttttccttacccatTTTGTAATTAAAACTTAGATTAGATTGCT | P*_TEF1_* |
| R03 | agcaatctaatctaagttttaattacaaaATGGGTAAGGAAAAGACTCACGTTTC | Neo^r^ |
| R04 | gtgaatgtaagcgtgacataactaattacatgaTTAGAAAAACTCATCGAGCATCAAAT | Neo^r^ |
| R05 | atttgatgctcgatgagtttttctaaTCATGTAATTAGTTATGTCACGCTTACATTCAC | T*_CYC1_* |
| R06 | ctgcaggtcgacGCAAATTAAAGCCTTCGAGCGTCCC | T*_CYC1_* |
| R07 | gaaggctttaatttgcgtcgacGAATCCTTACATCACAC | P*_TEF1_* |
| R08 | ggaaaggaacgggtgccatTTTGTAATTAAAACTTAGATTAGATTGC | P*_TEF1_* |
| R09 | gcaatctaatctaagttttaattacaaaATGGCACCCGTTCCTTTCC | *GLR1* |
| R10 | gtgccaagcttgcatgcctgcagAGACATAAAAAACAAAAAAATTACTTGCGAGTCGGGTATTTAC | *GLR1* |
| qGLR1-F | CACATGATCAAGACGGGTAT |  |
| qGLR1-R | GATCTTTTCACCCTCCTTGG |  |
| qACT1-F | GTTGGAGACGAAGCTCAG |  |
| qACT1-R | ATCTTTTCCATGTCATCCC |  |

Table S2. Differentially expressed genes and their encoded proteins

| Gene names | Up/Down | Encoded proteins |
| --- | --- | --- |
| gene_152829 | Up | hypothetical protein C6P46_002021 |
| gene_191597 | Up | sulfur metabolite repression control protein |
| gene_625116 | Up | glutathione reductase |
| gene_234300 | Up | hypothetical protein C6P46_004931 |
| gene_266854 | Up | hypothetical protein |
| gene_279309 | Up | zinc finger, GATA-type protein |
| gene_28436 | Up | hypothetical protein C6P46_006712 |
| gene_312 | Up | acyl-CoA N-acyltransferase |
| gene_539390 | Up | AMP phosphotransferase |
| gene_149520 | Down | hypothetical protein B0A53_02868 |
| gene_191322 | Down | hypothetical protein |
| gene_501084 | Down | hypothetical protein |
| gene_503423 | Down | hypothetical protein |


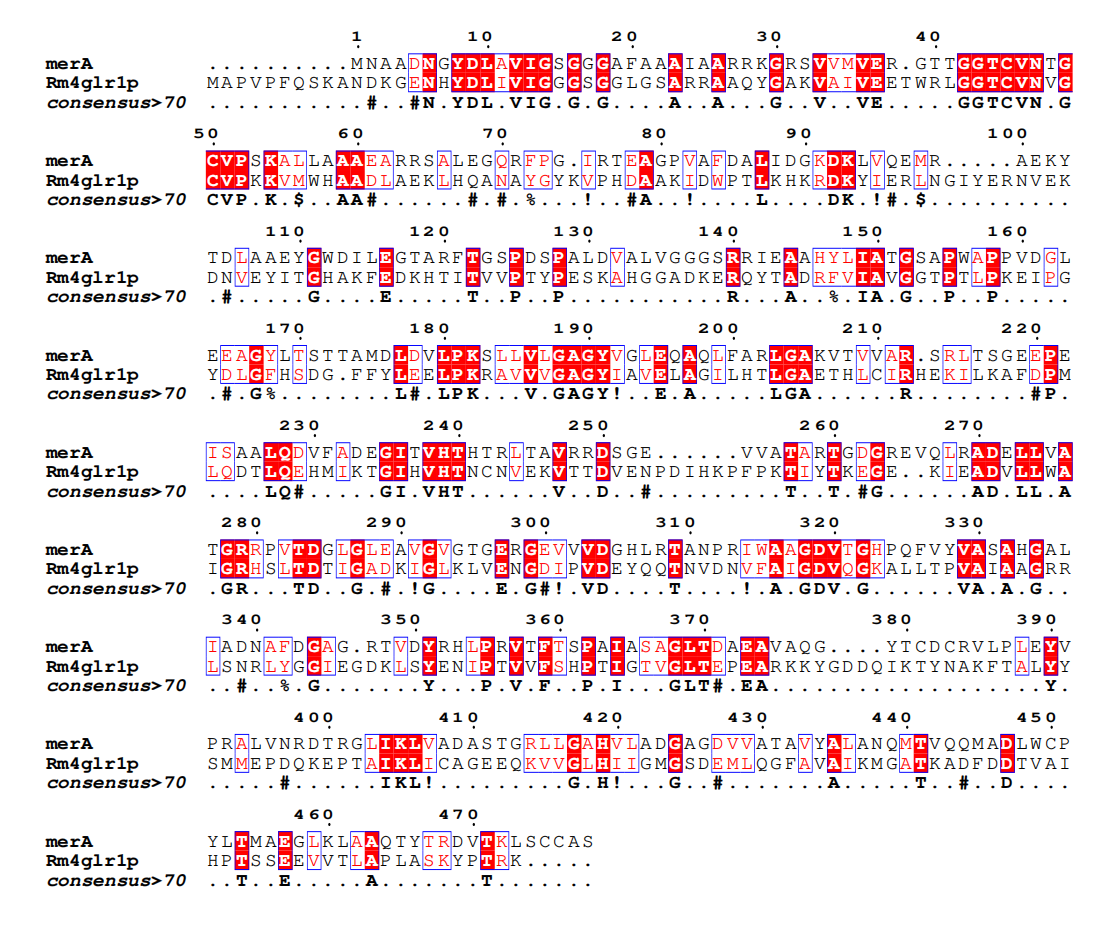


Figure S1. Sequence alignment of merA and Glr1p

Table S3. Binding site list

|  | Binding site |
| --- | --- |
| 1 | I23 |
| 2 | G24 |
| 3 | G26 |
| 4 | S27 |
| 5 | G28 |
| 6 | V46 |
| 7 | E47 |
| 8 | E48 |
| 9 | T49 |
| 10 | G54 |
| 11 | T55 |
| 12 | C56 |
| 13 | V59 |
| 14 | G60 |
| 15 | C61 |
| 16 | K64 |
| 17 | K65 |
| 18 | G128 |
| 19 | H129 |
| 20 | A130 |
| 21 | A167 |
| 22 | V168 |
| 23 | G169 |
| 24 | S187 |
| 25 | D188 |
| 26 | Y207 |
| 27 | I208 |
| 28 | S303 |
| 29 | I307 |
| 30 | G339 |
| 31 | D340 |
| 32 | L346 |
| 33 | L347 |
| 34 | T348 |
| 35 | P349 |
| 36 | A351 |
| 37 | V379 |
| 38 | F381 |

Table S4. Effect of *GLR1* on methylmercury resistance of *R.mucilaginosa*

|  | Rm4 | Rm4-GLR1 |
| --- | --- | --- |
| MIC (mg/L) | 3 | 5 |
| MLC (mg/L) | 6 | 7 |

1. * [↑](#footnote-ref-2)
2. * [↑](#footnote-ref-3)
